# A Machine Learning Approach That Measures Extracellular pH Within an In Vivo Tumor Model Using acidoCEST MRI

**DOI:** 10.1002/nbm.70379

**Published:** 2026-08-26

**Authors:** Alia Khaled, Chetan B. Dhakan, Lucas B. McCullum, Jorge de la Cerda, F. William Schuler, Tianzhe Li, Mark D. Pagel

**Affiliations:** ^1^ Department of Cancer Systems Imaging University of Texas MD Anderson Cancer Center Houston Texas USA; ^2^ Department of Medical Physics University of Wisconsin Madison Wisconsin USA; ^3^ Department of Radiation Oncology University of Nebraska Omaha Nebraska USA

**Keywords:** CEST MRI, iopamidol, machine learning, molecular imaging, pH imaging, tumor acidosis

## Abstract

Quantitative mapping of extracellular tumor pH (pHe) using acidoCEST MRI offers a method to characterize the tumor microenvironment. Conventional analysis of acidoCEST MRI relies on fitting the Bloch–McConnell equations to experimental CEST spectra. However, this “Bloch fitting” method is computationally intensive. Recent work has demonstrated that machine learning can accurately predict pH from CEST spectra of phantoms containing iopamidol, providing an alternative to Bloch fitting for acidoCEST MRI analysis. In this study, we evaluated the ability of machine learning models to estimate extracellular tumor pHe using acidoCEST MRI in a 4T1 murine breast cancer model. Thirty‐eight tumor‐bearing mice were imaged using a CEST‐FISP acquisition, and CEST spectra were extracted along with T_1_, T_2_, B_1_, and B_0_ maps. Pixel‐wise pH values derived from Bloch fitting served as reference values. We trained and tested three machine learning models: (1) random forest regression (RFR) using CEST spectra and T_1_, T_2_, B_1_, and B_0_ maps; (2) RFR using only CEST spectra; and (3) a convolutional neural network (CNN) using only CEST spectra. The RFR model using only CEST spectra achieved the best performance, with mean absolute percentage errors of 0.29% (training) and 0.79% (testing), corresponding to 0.020 and 0.054 pH units, respectively. Spatial pHe maps generated by the RFR methods closely matched those from Bloch–McConnell fitting, whereas CNN‐derived maps showed compressed pH ranges and reduced correlation with reference pH values. These findings demonstrate that RFR provides a computationally efficient alternative to Bloch fitting for in vivo acidoCEST MRI.

## Graphical abstract

Machine learning models, particularly RFR (random forest regression), provide accurate and computationally efficient tumor pHe estimation from acidoCEST MRI without requiring auxiliary parametric maps. RFR achieved comparable performance to traditional Bloch fitting (RMSE: 0.020 vs. 0.054 pH units), whereas CNN models showed reduced spatial coherence. The faster analysis with RFR versus. Bloch fitting supports clinical translation of ML‐based pH imaging for cancer diagnoses and therapy monitoring.

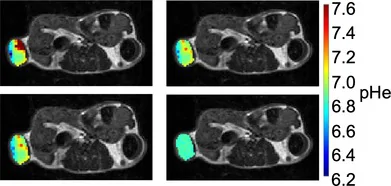

**Abbreviations**
acidoCEST: a version of CEST MRI that measures in vivo pHe
CEST: chemical exchange saturation transfer
CNN: convolutional neural network
FISP: fast imaging with steady state precession
MAPE: mean absolute percentage error
ML: machine learning
MRI: magnetic resonance imaging
MSME: multislice multiecho
pHe: extracellular pH
RARE: rapid acquisition with relaxation enhancement
RAREVTR: rapid acquisition with relaxation enhancement with variable time of recovery
RFR: random forest regression
ROI: region of interest
WASSR: water saturation shift referencing

## 1 Introduction

An acidic tumor microenvironment is a defining feature of solid malignancies [1, 2, 3]. Extracellular tumor pH (pHe) typically ranges from 6.4 to 7.0 (at 0.1–1 mm spatial resolution; finer spatial resolutions may exhibit even lower pHe values). These acidic pHe values are significantly lower than the tightly regulated pHe of normal tissues at pHe 7.2–7.4. Tumor acidosis is a consequence of biological processes such as elevated glycolysis and poor perfusion [4, 5]. This extracellular acidification plays an active role in enhancing extracellular protease activity that degrades the extracellular matrix remodeling to promote invasion and metastasis, modulating weak‐base drug uptake and ion trapping, and causing resistance to immunotherapy [6]. As a result, noninvasive quantification of pHe has emerged as an important objective for characterizing tumor aggressiveness, predicting and monitoring response to therapy, and guiding the development of pH‐sensitive therapeutics [7, 8].

Chemical exchange saturation transfer (CEST) MRI provides a powerful imaging platform for pH mapping by exploiting the pH dependence of proton exchange rates between water and endogenous metabolites or exogenous agents [9, 10]. Among exogenous agents, the contrast agent iopamidol is widely used for acidoCEST MRI, a specific protocol that uses CEST MRI, because its two amide proton pools at 4.2 and 5.6 ppm have base‐catalyzed exchange properties that are highly sensitive to physiologic tumor pHe [11, 12, 13]. A ratio of the % CEST signal from each proton pool can be used to estimate pH without dependence on the concentration of the agent. A more sophisticated method relies on iteratively adjusting the parameters of modified Bloch–McConnell equations that include pH as a parameter to fit an experimental CEST spectrum (also known as a Z spectrum) of iopamidol, known as “Bloch fitting” [14]. This analysis method simultaneously models chemical exchange rates, agent concentration, B_1_ and B_0_ magnetic field inhomogeneities, and T_1_ and T_2_ relaxation time constants of water (referred to as T_1_ and T_2_ for the remainder of this report). The estimated exchange rates are mostly independent from the other parameters, and these exchange rates are correlated with pH [15].

However, Bloch fitting requires nonlinear optimization of many parameters, making this iterative fitting method computationally intensive and time‐consuming [16]. In addition, CEST spectra can have low signal‐to‐noise considering that the % CEST effect is typically only a < 5% decrease of the MR signal. CEST MR images are acquired with little or no signal averaging to maintain a practical scan time, so that the signal‐to‐noise cannot benefit from extensive signal averaging. As with other iterative nonlinear fitting methods, the precision of pH estimates from Bloch fitting is directly dependent on signal‐to‐noise.

Recent advances in machine learning (ML) have introduced new approaches to accelerate quantitative MRI analyses, including applications in diffusion MRI, tissue segmentation, synthetic contrast generation, parameter mapping, and quantitative susceptibility mapping [17, 18, 19, 20]. In the context of CEST MRI, ML‐based analysis has been explored in preliminary studies, demonstrating the potential to learn nonlinear spectral–pH relationships directly from CEST spectra without explicit physical modeling [21, 22]. These methods have the advantage of bypassing complex differential‐equation fitting and instead use data‐driven mappings that are inherently more tolerant of noise and experimental variability. Specifically, our prior phantom work showed that random forest regression (RFR) achieved highly accurate pH predictions from iopamidol spectra, eliminating the need for complex fitting procedures [23]. However, although promising, ML approaches have not been comprehensively evaluated in in vivo acidoCEST MRI, where in vivo tissues may limit model generalizability.

In this study, we investigated the capability of ML models to estimate tumor pHe from in vivo acidoCEST MRI using a 4T1 breast cancer model. We used pixelwise pHe values from Bloch fitting as the reference standard when developing our ML models. We compared three approaches: (1) RFR using CEST spectra and parametric maps of T_1_, T_2_, B_1_, and B_0_ magnetic field inhomogeneities; (2) RFR using CEST spectra alone; and (3) a convolutional neural network (CNN) using CEST spectra alone. We compared our parametric maps of tumor pHe from each of these ML methods with tumor pHe maps derived from Bloch fitting as the reference standard. Our primary goals were to determine if ML can accurately and reproducibly estimate tumor pHe in vivo; whether parametric maps of T_1_, T_2_, B_1_, and B_0_ improve or degrade ML prediction accuracy; and whether ML generates spatially consistent pHe maps that reproduce physiologic gradients observed in pHe maps derived from Bloch fitting.

## 2 Experimental

### 2.1 Tumor Xenograft Animal Model

Thirty‐eight female BALB/c mice, 6–8 weeks old, were purchased from Jackson Labs Inc., Bar Harbor, ME, United States. The mice were subcutaneously implanted with 2.0 × 10^6^ 4T1 cells in both flanks with a 27‐gauge insulin syringe. When mice showed signs of palpable tumor with an ~5 mm diameter, mice were scanned for MRI acquisition. Tumor volume (V) was estimated using the following formula, *V* = *A* × *B* ^2^ / 2, where *A* and *B* are the longest and the shortest diameters, respectively [24]. Following MRI acquisition, mice were transferred to a biosafety hood and maintained under continuous anesthesia. Tumor pHe was measured in vivo at three locations along the tumor using a pH microsensor (Precision Sensing GmbH, Regensburg, Germany). Mice were subsequently euthanized immediately after pH measurements.

### 2.2 MRI Acquisitions

A mouse was initially anesthetized with 3%–5% isoflurane in 100% oxygen gas and subsequently maintained under 1.5%–2.0% isoflurane anesthetic for the duration of the MRI acquisition session. During the preparation phase, the mouse was placed on a warming pad to maintain body temperature under anesthetic. A catheter was constructed from a 27‐g needle, and PE10 tubing was inserted into the tail vein, which was then connected to a sterile syringe containing 200 μL of 976 mM iopamidol in water (IsoVue‐370, Bracco Diagnostics Inc., Princeton, NJ, United States). The mouse was secured to a customized cradle, and the cradle was inserted into a 7 T magnet of a BioSpec MRI scanner with a 35‐mm diameter quadrature transceiver coil (Bruker BioSpin Inc., Billerica, MA, United States). The base of the cradle contained a water circulation pad with warm water to maintain body temperature. Furthermore, the body temperature was accurately regulated at 37°C ± 1.0°C using a rectal temperature probe and warm air that was regulated by an automated feedback system (SA Instruments Inc., Stony Brook, NY, United States).

Detailed lists of MRI acquisition parameters are available in the Supporting Information. Prior to CEST MRI acquisition, an anatomical RARE MRI scan was performed to initially survey tumor positioning across the imaging volume. A second RARE MRI acquisition with a single‐slice scan was acquired to confirm an optimal slice location for the subsequent single‐slice MRI acquisitions. A RAREVTR MRI acquisition was performed to obtain a T_1_ map; a MSME MRI acquisition was performed to obtain a T_2_ map; a double‐angle gradient echo MRI acquisition was performed to obtain a B_1_ map, and a WASSR MRI acquisition was performed to obtain a B_0_ map [25, 26, 27, 28]. To perform acidoCEST MRI, a series of four CEST‐FISP MRI scans were acquired, and then, 200 μL of 370 mg of iodine per mL of iopamidol (Isovue‐370, Bracco Imaging S.p.A., Milan Italy) was intravenously injected [29]. The injection line was then connected to an infusion pump which delivered 400 μL/h if the same contrast agent for the remainder of the scan period. The mouse was then scanned with CEST‐FISP MRI for six repetitions.

A region of interest (ROI) was manually determined for each mouse, which was used to extract MR signal amplitudes for CEST, T_1_, T_2_, B_1_, and B_0_ analyses. Each ROI consisted of approximately 48 pixels. Technical details about image analyses to obtain T_1_, T_2_, B_1_, and B_0_ maps are listed in the Supporting Information. A description of the Bloch fitting method to obtain pHe maps is also available in the Supporting Information.

A phantom of 20‐mM iopamidol in water with a volume of 0.6 mL was also scanned with CEST‐FISP MRI using the same acquisition parameters used for in vivo CEST‐FISP MRI. This CEST spectrum of a phantom was compared to the in vivo CEST spectrum to verify that CEST was generated at 4.2 and 5.6 ppm in vivo.

### 2.3 ML Model Development and Training

**FIGURE 1:**
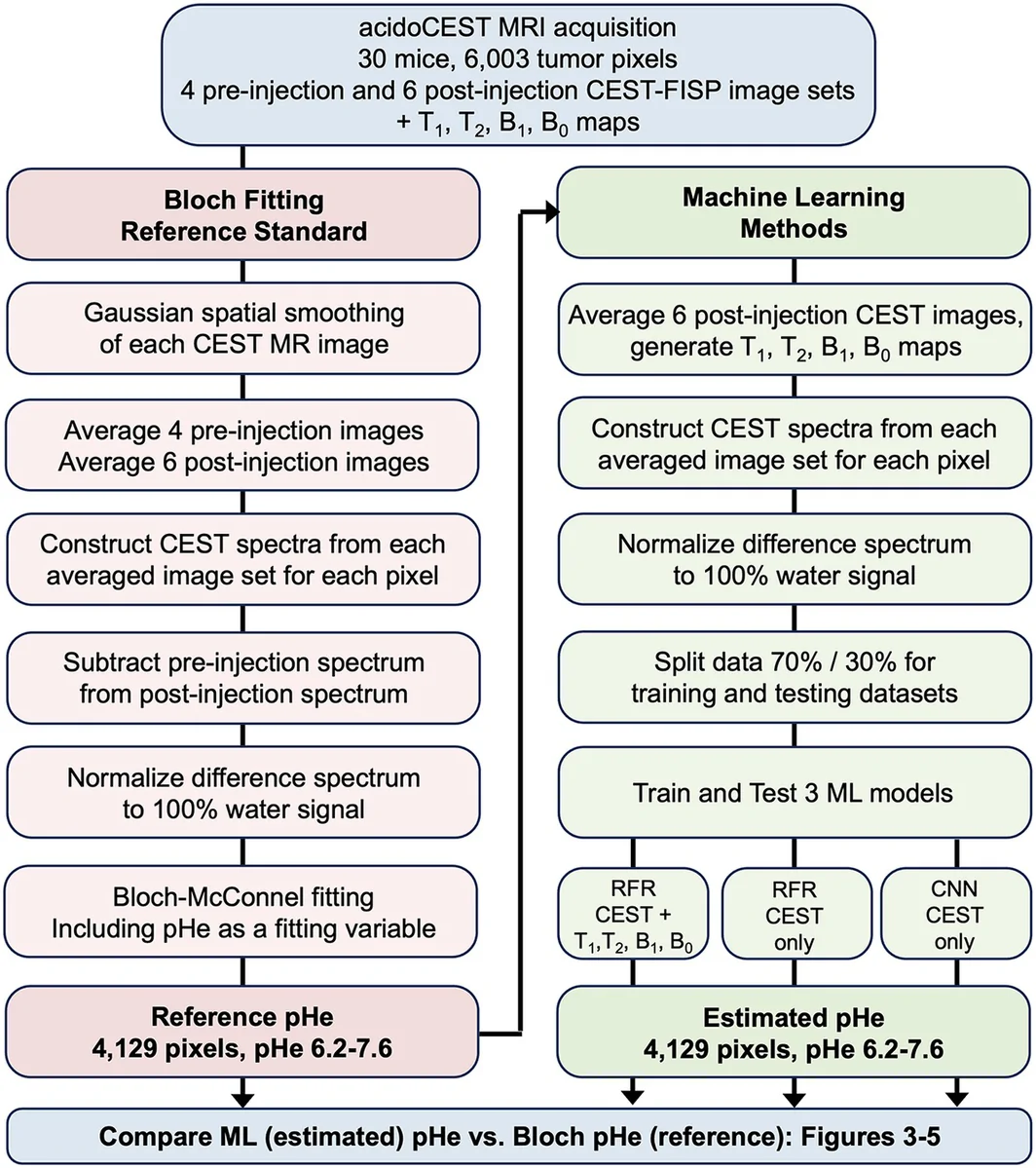
Machine learning workflow for pHe estimation from acidoCEST MRI. acidoCEST MRI data from 38 mice (6003 pixels total) were used to generate reference pHe values via Bloch fitting (left), which subtracts the averaged preinjection CEST spectrum from the averaged postinjection CEST spectrum to isolate the iopamidol‐specific signal prior to fitting. In parallel, three machine learning models (right) were trained on the raw post‐injection CEST spectra (with or without T_1_, T_2_, B_1_, and B_0_ maps) after filtering to the pH range 6.2–7.6 (4129 pixels) and a 70/30 train/test split. Predicted pHe values from each model were compared against the Bloch fitting reference standard (Figures 3, 4, 5).

We used pixelwise pHe values from Bloch fitting as the reference standard when developing our ML models (Figure 1). CEST spectra that have very low or no CEST signal with saturation at 4.2 and 5.6 ppm cause the Bloch fitting process to essentially fit noise, resulting in highly erroneous pHe estimates that are much lower than pH 6.2 and much higher than pH 7.6. Therefore, we only retained pixels with pHe estimates between 6.2 and 7.6, which discarded 1874 pixels (31%) and retained 4129 pixels (69%) with pHe values for use as a reference standard when developing our ML models. Pooling the pixels from all tumors assumes that all tumors have the same tissue characteristics. This assumption was reasonable considering that all tumors were prepared by using the same cell type and injection conditions, using the same mouse strain, sex, and age, and only allowing the tumors to reach the same ~5 mm diameter prior to imaging.

The RFR model and the CNN model (Figure S1) were implemented in scikit‐learn 1.5.2 and keras 2.10.0 on Python 3.10.8 [30, 31]. The CEST spectrum used as input for all three models was constructed from the average of the six postinjection acidoCEST‐FISP acquisitions and normalized to 100% water signal at the −100 ppm saturation frequency that served as a control. The spectra were not smoothed to further improve signal to noise. This process differs from the Bloch fitting procedure used to generate reference pHe values, which used Gaussian spatial smoothing and subtracted the average preinjection spectrum from the average postinjection spectrum to isolate the iopamidol‐specific signal (see Section S3). The CEST spectrum and the T_1_, T_2_, B_1_, and B_0_ values for each pixel were used as features for the first RFR model. The CEST spectra alone were used as features to the second RFR model and the CNN model. Data were split into two sets for training (70%) and testing (30%) sets at the pixel level using scikit‐learn's train_test_split (random_state = 42), pooling pixels across all tumors. The mean absolute percentage error (MAPE) and the pH unit error were used to evaluate the performance of each of the models.

The RFR model used an ensemble approach with multiple decision trees to predict pHe values. Threefold cross‐validation was used to optimize hyperparameters including the number of trees (200, 300, or 500) and maximum tree depth (10, 20, 40, or unlimited), using GridSearchCV with the coefficient of determination (*R* ^2^) as the optimization criterion; the split‐quality criterion for individual trees was the scikit‐learn default (mean squared error). The selected model using CEST spectra with T_1_, T_2_, B_1_, and B_0_ maps used 200 trees with a maximum depth of 40; the model using CEST spectra alone used 500 trees with a maximum depth of 40 (both with random_state = 42). RFR input features were not scaled prior to training. Feature importance was assessed for both RFR models to identify the most relevant saturation frequencies and MRI parameters for pH prediction.

The CNN architecture consisted of four convolutional blocks, each containing a 1D convolutional layer with 64 filters, kernel size of 3, and ReLU activation, followed by a max pooling layer with pool size of 2. Following the convolutional blocks, the network included a flatten layer to convert the 2D feature maps to 1D, a dropout layer with rate of 0.2 to prevent overfitting, and two fully connected layers with 64 units and ReLU activation for the first layer and one unit with linear activation for the final output layer. The network was trained using the Adam optimizer (learning rate = 0.01) with a batch size of 32, with mean absolute error as the loss function, for up to 200 epochs with early stopping (patience of five epochs, monitored on a 20% validation split of the training data). Full training code and data are publicly available (Data Availability Statement).

## 3 Results

### 3.1 In Vivo Imaging Characteristics

**FIGURE 2:**
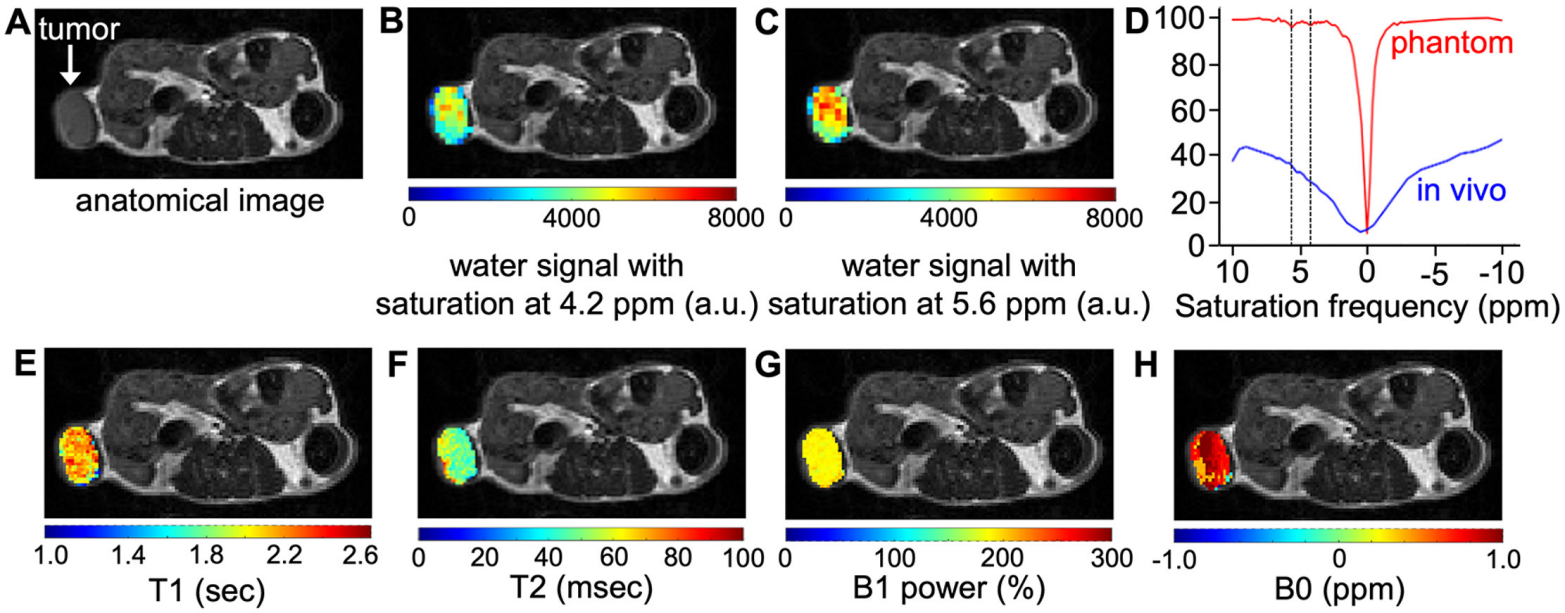
Multiparametric MR imaging of a tumor‐bearing mouse. (A) The anatomical image shows the tumor location. (B) CEST maps of % water signal at 4.25 ppm and (C) at 5.5 ppm. (D) A CEST spectrum of in vivo tissue (red) compared with a phantom (blue). The vertical dashed lines indicate values at 4.2 and 5.6 ppm. (E) A T_1_ map, (F), T_2_ map, (G), B_1_ map, and (H) B_0_ map.

All mice survived through the multiparametric MRI acquisitions, which required less than 1 h including prescan preparation and postscan recovery. The high‐quality anatomical images showed clear tumor localization (Figure 2A). Each parametric map of the tumor was overlaid on the anatomical image. The CEST signal amplitude maps at 4.25 ppm (Figure 2B) and 5.5 ppm (Figure 2C) showed tumor heterogeneity. The CEST spectrum constructed from the CEST images showed decreased % water signal at 4.2 and 5.6 ppm in the phantom and the in vivo tumor (Figure 2D). Other parametric maps showed spatial heterogeneity in T_1_ (Figure 2C), T_2_ (Figure 2D), B_1_ (Figure 2E), and B_0_ (Figure 2F) values throughout the tumor.

### 3.2 Comparison of ML Methods

#### 3.2.1 pH Classification Results

**FIGURE 3:**
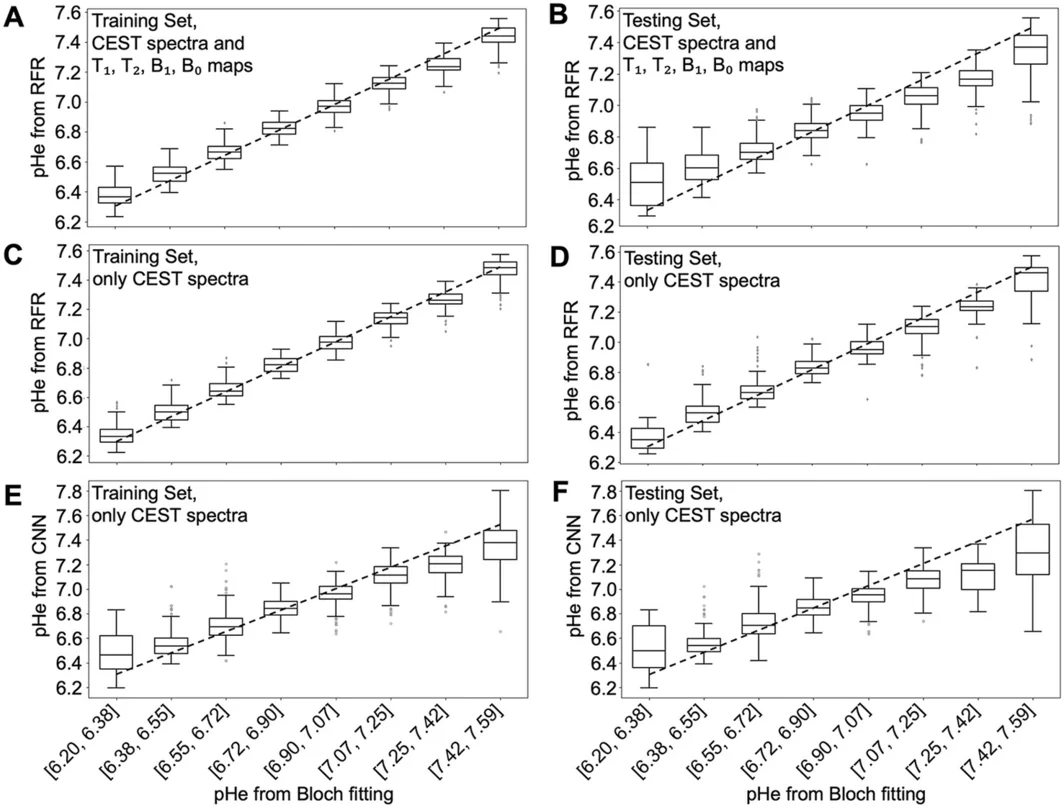
Correlation of pHe measurements between Bloch fitting and ML methods using box‐and‐whisker plots. These plots show pHe distributions for training (left column) and testing (right column) datasets using (A, B) RFR using CEST spectra and T_1_, T_2_, B_1_, and B_0_ maps; (C, D) RFR using only CEST spectra; (E, F) CNN using only CEST spectra. This graph shows ranges of pHe values from Bloch fitting (e.g., pHe 6.20–6.38 for the left‐most box‐and‐whisker). For comparison, Figure S2 shows all 4129 data points. The dashed lines represent the line of identity. The box represents the middle 50% of the data distribution, with line within the box that represents the median value. The whiskers show the range of data values within 1.5 times the box height from the median.

We performed correlation analyses to compare pHe measurements with each ML approach versus pHe measurements from Bloch fitting for both training and testing datasets (Figures 3 and S2). Figure S2 shows all 4129 datapoints, but most datapoints are overlapped, causing the visualization of the data to be challenging. Therefore, Figure 3 shows small ranges of pHe values from Bloch fitting, which allows for better visualization of the accuracy and precision of the correlation analyses. These analyses evaluated an RFR model with CEST spectra and T_1_, T_2_, B_1_, and B_0_ maps (Figure 3A,B); RFR with CEST spectra only (Figure 3C,D); and a CNN with CEST spectra only (Figure 3E,F).

For the training datasets, all three models demonstrated strong correlations between predicted pHe from the ML models and reference pHe values from Bloch fitting. The pHe values from the RFR model using only CEST spectra showed the best accuracy throughout the entire pHe range (closely aligned with the line of unity throughout the pHe range in Figure 3C) while also exhibiting the highest precision (small standard deviations as shown by the box heights in Figure 3C). The RFR model using all features maintained a good accuracy in estimating pHe except at the extremes of the pHe range (Figure 3C). The CNN model exhibited the weakest correlation and the lowest precision for estimating pHe, suggesting potential overfitting to training data characteristics (Figure 3E).

The testing datasets revealed similar accuracies for each model but with increased imprecision in estimating pHe compared to training datasets (Figure 3B,D,F). The RFR model using only CEST spectra maintained the best accuracy and best precision. The RFR model using all features showed a good accuracy but with decreased precision especially at the extremes of the pHe range. The CNN model demonstrated the poorest accuracy and precision, particularly for samples with pH values below 6.5 and above 7.3.

These correlation analyses demonstrate that the RFR model, particularly when using only CEST spectra, provides superior accuracy and precision for pH estimation from acidoCEST MRI data compared to the CNN model. The consistency between training and testing datasets for the RFR models indicates minimal overfitting and excellent model generalization.

#### 3.2.2 Spatial pH Mapping Performance

**FIGURE 4:**
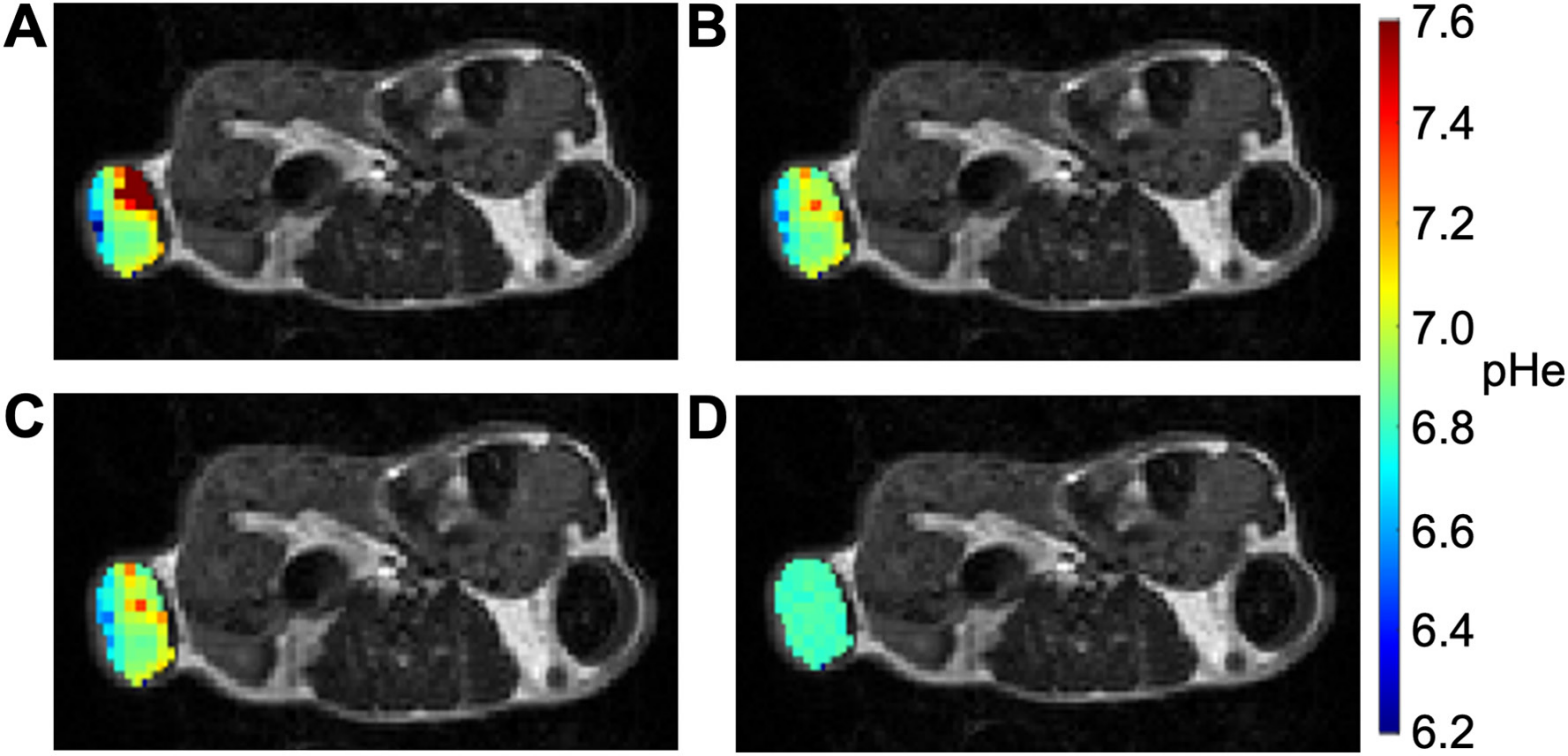
Tumor pHe maps from different analysis methods. Parametric pH maps generated using (A) Bloch fitting, (B) RFR using all features (CEST spectra, T_1_, T_2_, B_1_, and B_0_), (C) RFR using only CEST spectra, and (D) CNN using only CEST spectra.

A representative pHe map from Bloch fitting showed clear acidic regions (blue‐green, pHe ~ 6.6–6.8) in portions of the tumor and more alkaline regions (yellow‐orange, pHe ~ 7.1–7.3) in adjacent areas, consistent with expected tumor microenvironmental heterogeneity (Figure 4A). The RFR model incorporating all features (Figure 4B) and the RFR model using only CEST spectra (Figure 4C) produced pHe maps with spatial heterogeneity patterns most closely matching the pHe map from Bloch fitting, including distinct acidic and alkaline regions with similar boundaries and intensity distributions for this representative example. The similarity of the pHe maps from both RFR methods indicated that using only CEST spectra was sufficient to produce the pHe map. The CNN model using only CEST spectra (Figure 4D) generated a pHe map with reduced dynamic range and most values concentrated within pH 6.8–7.2. This compressed pH distribution suggests the CNN model underestimates pH variations within tumors. Other pHe maps generated from all four methods showed the same conclusions as this representative example in Figure 4. Quantitatively, all three ML methods correctly identified the general acidic nature of the extracellular tumor microenvironment, showing pHe < 7.0. However, the RFR method clearly outperformed the CNN method.

**FIGURE 5:**
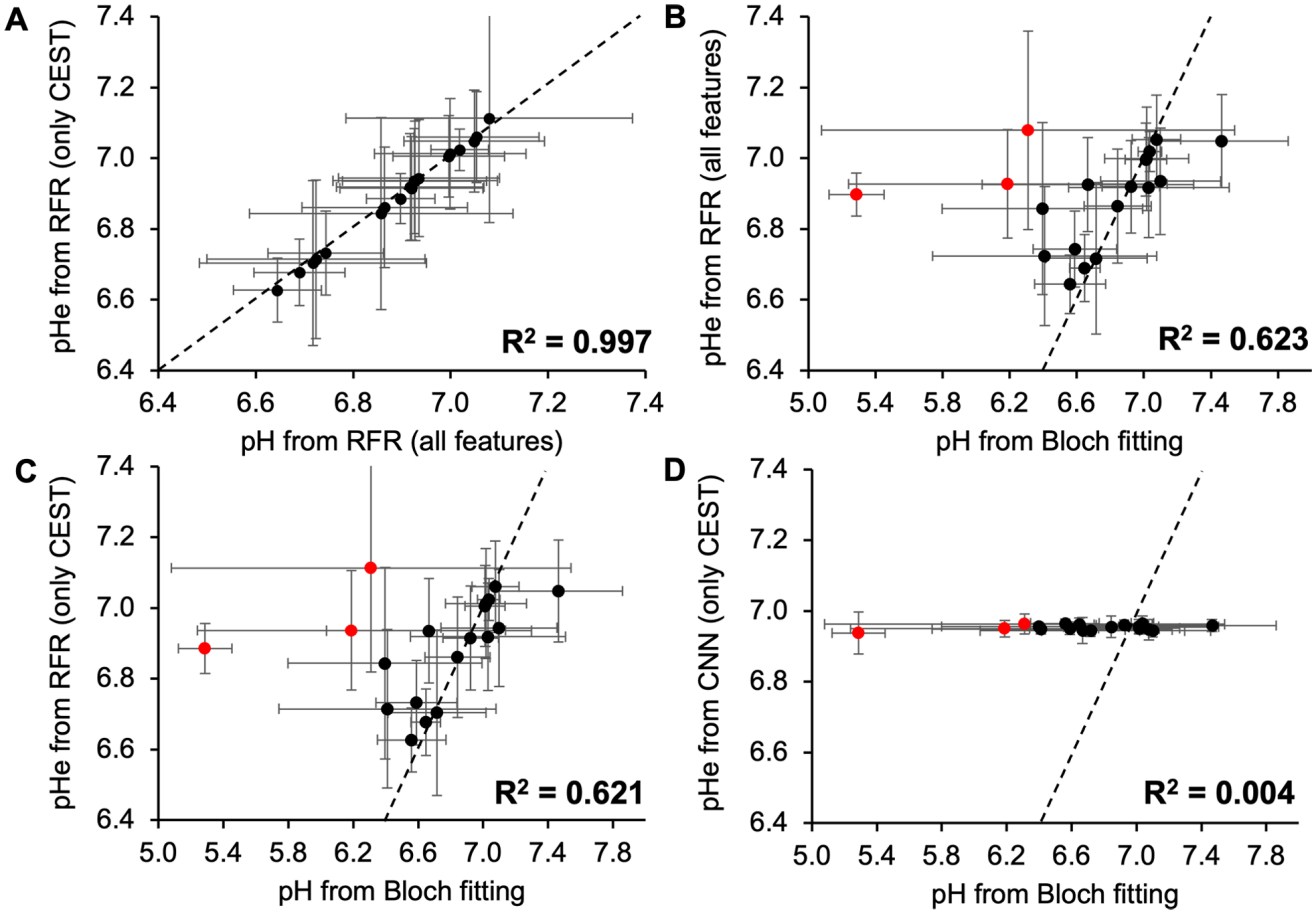
Comparison of pHe values from different analysis methods. (A) A scatter plot with correlation statistics comparing the pHe values from RFR with all features versus RFR with CEST only. Scatter plots with correlation statistics comparing the pHe values from Bloch fitting versus (B) RFR using all features, (C) RFR using only CEST spectra, and (D) CNN using only CEST spectra. Red data points represent pHe values from Bloch fitting that are lower than pHe 6.2, which is the lower limit of this fitting method. The red data points were not included in the calculation of the *R* ^2^ correlation coefficients.

These results were further substantiated through graphical representations (Figure 5). A comparison of the pHe values estimated from RFR using all features or only CEST spectra showed an outstanding *R* ^2^ value of 0.997 and near‐perfect concordance along the line of identity. This similarity from Figure 5A matched the similarities between the spatial maps in Figure 4B,C. This result indicates that the T_1_, T_2_, B_1_, and B_0_ parametric maps provide no improvement in pHe estimates, and the CEST spectra contain the primary information about pHe.

The pHe values from Bloch fitting versus the RFR method revealed good correlations but with larger differences at the extremes of the pHe range. These results from Figure 5B,C matched the similarities between the spatial maps in Figure 4A versus Figure 4B and Figure 4A versus Figure 4C. The pHe values from Bloch fitting versus the CNN method in Figure 5D showed no correlation, which matched the comparison of the spatial maps between these analysis methods in Figure 4A versus Figure 4D, which further demonstrated the limited performance of the CNN approach for estimating pHe.

#### 3.2.3 Quantitative Performance Metrics

We evaluated the quantitative performance of the three ML models by evaluating the differences of each pHe measurement from a ML method versus the pHe estimated from Bloch fitting (Table 1). Based on the standard deviation of these differences, the RFR model using only CEST spectra produced the best precision, with a remarkably low standard deviation of 0.054 pHe units with the testing dataset. The RFR model using all features also achieved excellent precision with a standard deviation of 0.094 pH units with the testing dataset. However, this result suggested that inclusion of all parametric maps in the RFR modeling may introduce minor overfitting. Feature importance analysis of both RFR models showed that importance was broadly distributed across the saturation frequency range rather than concentrated at the 4.2‐ and 5.6‐ppm amide‐proton offsets (Figure S3).

**TABLE 1:** Performance comparison of machine learning models for pHe estimation.

Model	Features	Standard deviation			
		Mean absolute percentage		pHe unit	
		Train	Test	Train	Test
RFR	CEST spectra, T_1_, T_2_, B_1_, B_0_	0.51%	1.37%	0.035	0.094
RFR	CEST spectra	0.29%	0.79%	0.020	0.054
CNN	CEST spectra	1.31%	1.70%	0.090	0.117

As an additional validation, both RFR models were retested after randomly permuting pHe labels prior to training; both permuted models performed markedly worse than the trained models, including negative *R* ^2^ values on the test set (Table S1), confirming that model accuracy reflects a genuine learned relationship rather than chance association.

In contrast, the CNN model using only CEST spectra demonstrated substantially inferior precision, with a standard deviation of 0.117 pH units for the testing dataset. This result indicated that a CNN architecture was suboptimal for this specific application of pHe estimation from CEST spectra, despite the theoretical advantage of CNN for complex pattern recognition.

#### 3.2.4 Validation Against Reference pH Measurements

**FIGURE 6:**
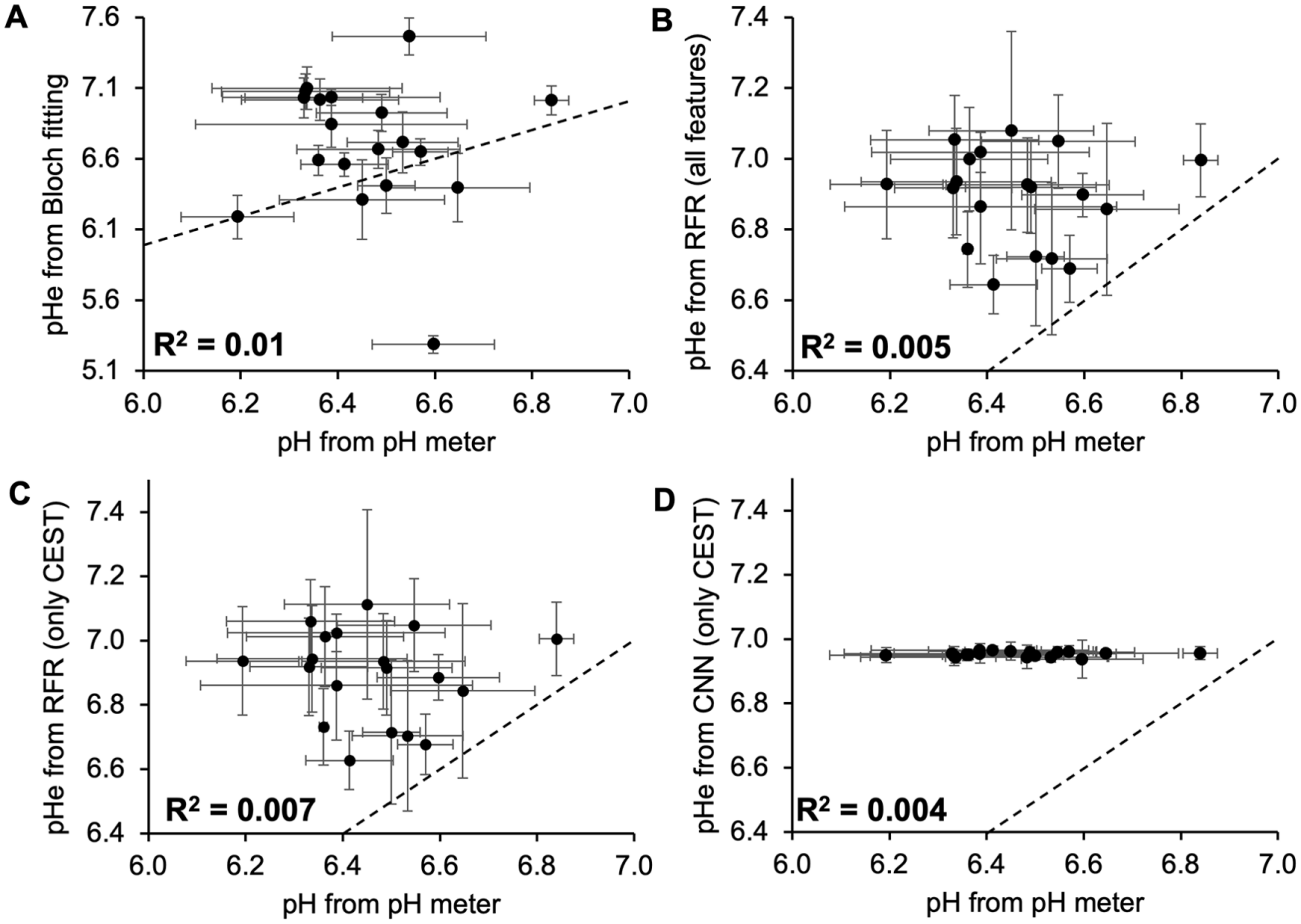
Comparison of pHe values from different analysis methods versus an in vivo pH meter. Scatter plots with correlation statistics comparing the tumor pH values from an in vivo pH meter versus tumor pHe values from (A) Bloch fitting, (B) RFR using all features, (C) RFR using only CEST spectra, and (D) CNN using only CEST spectra. The average and standard deviation of all pixels of an individual tumor were compared to the average and standard deviation of all pH microelectrode measurements for each individual tumor.

We compared tumor pHe estimates from each of the analysis methods with tumor pH measurements made with an in vivo pH microelectrode (Figure 6). These results showed poor correlations between pHe values from acidoCEST MRI and pH values from the microelectrode. This lack of a correlation may be attributed to the fact that tumor pHe is not the same as tumor pH because pHe is the pH in only the extracellular, extravascular tumor microenvironment whereas pH is measured from a mixture of all tumor spaces. Furthermore, a pH microelectrode is an invasive measurement technique, and tumor tissue damage may alter pH in the tumor. As another observation, the standard deviations of the pH measurements made with an in vivo pH microelectrode (horizontal error bars in Figure 6) are much larger than the standard deviations of the pHe measurements from acidoCEST MRI (vertical error bars in Figure 6). This larger imprecision indicated that the pH microelectrode measurements were less reliable than estimates from acidoCEST MRI. Finally, this analysis showed that the pHe values estimated from CNN using only CEST spectra had a poor dynamic range.

## 4 Discussion

This study demonstrated that an ML approach can provide accurate and computationally efficient estimation of tumor pHe from acidoCEST MRI data. More specifically, our findings indicate that CEST contains sufficient pH‐sensitive information for accurate pH estimation without explicit physical modeling, supporting the feasibility of ML‐based approaches for rapid and reliable tumor acidoCEST MRI analysis. Our results show that the RFR model trained only on CEST spectra achieved pixelwise pHe values with the best accuracy and precision using pHe values from Bloch fitting as the reference. The CNN model achieved low training error but demonstrated increased variance and reduced spatial coherence in testing, suggesting potential overfitting to training data characteristics. Bie et al. [31] demonstrated that a CNN trained on simulated CEST spectra could classify discrete tumor tissue types. Our study applies both CNN and RFR models trained on experimental in vivo data to continuously quantify tumor pHe, demonstrating that RFR outperforms CNN for this regression task.

The primary advantage of ML‐based pH estimation lies in its computational efficiency [30]. Bloch fitting requires iterative nonlinear optimization for each pixel, which is time‐consuming and sensitive to initialization parameters. In contrast, the RFR model generates pHe estimates rapidly once trained, making it practical for near‐real‐time pHe mapping during imaging sessions. In addition, the RFR model does not require spatial or spectral smoothing, further simplifying the analysis. This computational advantage is particularly relevant for clinical translation, where rapid image reconstruction and immediate feedback are essential for treatment monitoring and adaptive therapy guidance [32, 33]. Furthermore, the RFR model only requires a postinjection CEST spectrum, which greatly simplifies the acidoCEST MRI acquisition protocol.

Our study revealed potential limitations of CNN approaches for CEST pH mapping. The CNN model demonstrated lower accuracy and precision in pHe estimates with the test dataset, suggesting overfitting to spectral noise patterns or spurious correlations. The increased complexity of CNN architectures compared to RFR may require substantially larger and more diverse training datasets to achieve robust generalization. These larger training sets could be provided by more experimental results or by a data augmentation method [30].

Our current in vivo studies match our previous in vitro studies that employed ML for analysis of acidoCEST MRI [23]. This in vitro study acquired 21,600 CEST spectra of 120 phantoms of iopamidol, along with T_1_, T_2_, B_1_, and B_0_ maps. Similar to our in vivo study, the RFR model showed the best performance for estimating pH relative to measurements using a calibrated pH meter among all ML models tested. However, the in vitro study showed an improvement when a B_0_ map was included with the CEST spectra for the analysis, whereas our in vivo study showed no improvement with including a B_0_ map. This difference may be due to the high B_0_ homogeneity in MRI studies that evaluate homogeneous phantoms relative to the lower B_0_ homogeneity encountered with heterogeneous tissues during MRI studies.

Both our previous in vitro study and our current in vivo study showed the best performance for estimating pH when the RFR model did not include a B_1_ map. This result was surprising because a B_1_ map is often considered to be essential for quantitative CEST measurements. Our results with the RFR model without a B_1_ map may indicate that experimentally measuring the B_1_ saturation power inherently contains inaccuracies and/or imprecisions that degrade the value of including B_1_ in ML methods for quantitative CEST measurements.

Feature importance analysis (Figure S3) showed that auxiliary MRI parameters (B_0_, B_1_, and T_2_) ranked among the highest importance features when available, despite their inclusion providing no measurable improvement in accuracy relative to the CEST‐only model (*R* ^2^ = 0.997, Figure 5A). We interpret this as evidence that field‐ and relaxation‐dependent information is already encoded within the shape of the raw, uncorrected Z‐spectrum, rather than these auxiliary parameters contributing independent information.

The same feature importance analysis also showed that both RFR models drew broadly on the entire Z‐spectrum rather than concentrating on the amide‐proton frequencies (4.2 and 5.6 ppm) conventionally associated with iopamidol's pH sensitivity. The CEST spectra used for model training and testing were derived from postinjection acquisitions without subtraction of the pre‐injection baseline spectrum. Therefore, some of the ML models' predictive power reflects endogenous CEST signals related to pHe in addition to the two CEST signals from iopamidol.

This result indicates that endogenous CEST spectra without iopamidol may be able to predict pHe using ML methods. However, to develop a ML method, the ground truth pHe values for each CEST spectrum used to train the ML method must be known. We used Bloch fitting of acidoCEST MRI that used iopamidol in this study, and therefore, the CEST signals of iopamidol are necessarily incorporated in our ML analysis methods. Another imaging method could be used to generate ground truth pHe values for training ML methods to estimate pHe from endogenous CEST spectra. This other imaging method would need to accurately and precisely measure tumor pHe (and not pHi or total pH) without an exogenous contrast agent that has exchangeable protons (i.e., does not generate CEST signals) and produce images that can be registered with endogenous CEST MR images with the same spatial resolutions (i.e., multimodality image registration is a nontrivial complication). To date, this type of gold standard method does not exist. Hence, acidoCEST MRI with iopamidol is currently the best way to train a ML method to measure tumor pHe from CEST MRI results.

As further support, a permutation test in which pHe training labels were randomly shuffled prior to RFR model fitting resulted in substantially degraded performance, including negative *R* ^2^ values on the held‐out test set (Table S1). This result confirmed that the trained models are learning a genuine relationship between the input spectra and pHe rather than fitting noise or exploiting incidental correlations introduced by the pixel‐level train/test split.

Our analyses of CEST spectra with ML methods are similar to other reports that have employed ML analyses of CEST MRI results. In particular, neural networks for analyzing CEST spectra have been used to predict the amide proton transfer effect in preclinical brain tumors [34]; to classify glutamine distribution in mouse models of Alzheimer's Disease [35]; to classify pancreatic tumor models that differ in hypoxic status [36]; to evaluate choline phospholipids in tumor models [37]; and to assess guanidine levels in tumor models [38]. Other reports have shown that ML can accelerate the analysis process, similar to the results from our study. For example, ML methods have been implemented to reduce computation time when employing line shape fitting methods to experimental CEST spectra [39]. Therefore, our work contributes to this growing field of applying ML to CEST MRI analysis [40]. Yet our studies are unique by demonstrating that ML can be applied to analyze CEST MR images with an exogenous CEST agent, whereas all previous studies have focused on the analysis of endogenous CEST MRI.

The comparison of our tumor pHe estimates from acidoCEST MRI was not correlated with tumor pH measurements made with an in vivo pH microelectrode (Figure 6). We have previously shown a relationship between acidoCEST MRI results and pH microelectrode measurements with a moderate correlation (*R* ^2^ = 0.72) that was similar to the correlation in our current study (*R* ^2^ = 0.62) [12]. Other studies that have used MRI methods to measure intracellular tumor pH (pHi) have shown moderate agreement with in vivo pH microelectrode measurements [41, 42]. Therefore, our current studies and these previous studies suggest that pH, pHe, and pHi can be different. Furthermore, as shown by our current studies and these previous studies, measurements with an in vivo pH microelectrode have large variabilities, which represent large heterogeneity in tumor acidosis within different tumor regions sampled by a microelectrode and/or large imprecision with the invasive microelectrode.

Several technical refinements could further improve ML‐based acidoCEST pH mapping. Physics‐informed neural networks (PINNs) that embed Bloch–McConnell equations as soft constraints could combine the flexibility of data‐driven learning with the theoretical rigor of physical models [43]. Attention mechanisms could identify the most informative spectral frequencies for pH estimation, potentially enabling acquisition protocols with fewer saturation offsets and reduced scan time [44]. Translation to human imaging at clinical field strengths (3T) will require validation across different scanner vendors, acquisition protocols, and patient populations [45, 46]. Bloch fitting with acidoCEST MRI is arguably one of the best CEST imaging methods for estimating tumor pHe values and therefore served as the best available reference standard for our study. However, like all experimental methods, this method has some inaccuracy (0.055 pH units) and imprecision (0.046 pH units) even under the best conditions. This variability in Bloch fitting may have affected the variability in our ML methods. Our study provides a springboard for these new methods and additional ML‐based approaches for mapping tumor pHe with acidoCEST MRI.

## 5 Conclusion

This study demonstrated that RFR provides an accurate and precise estimate of tumor pHe while providing a computationally efficient alternative to traditional Bloch fitting for tumor pHe estimation from acidoCEST MRI. By eliminating the need for auxiliary parametric maps and intensive nonlinear optimization, ML‐based approaches have the potential to accelerate clinical translation of pH imaging. The superior performance of RFR over CNN highlights the importance of model selection and the value of simpler architectures when training data are limited. Further validation in diverse clinical scenarios and integration with multiparametric imaging platforms will be important next steps toward realizing the full potential of acidoCEST MRI for precision oncology.

## Author Contributions

A.K., C.B.D., T.L., and M.D.P. designed the study as an extension of a previous study championed by T.L. F.W.S. developed the tumor model. C.B.D., L.B.M., and J.d.l.C. performed the imaging acquisitions. A.K. and C.B.D. performed the image analysis. A.K., C.B.D., and M.D.P. drafted the manuscript. All authors updated or reviewed the final manuscript.

## Funding

This work was supported by the National Institutes of Health (R01 CA231513).

## Conflicts of Interest

The authors declare no conflicts of interest.

## Supporting information


**Table S1:** Permutation (label‐shuffling) test for the RFR models. Each RFR model was retrained with identical hyperparameters and features, but with pHe labels randomly permuted across training pixels prior to fitting (“Permuted” rows), and evaluated against the true, unpermuted test‐set labels. Both permuted models performed substantially worse than the corresponding trained models (“Real” rows), including negative R^2^ values (worse than predicting the mean pHe value), confirming that the trained models' accuracy reflects a genuine learned relationship between CEST spectral features and pHe rather than chance association or overfitting.

**Figure S1:** CNN architecture for acidoCEST MRI analysis. The 1D convolutional neural network consists of four convolutional layers (each with 64 filters, kernel size = 3, and ReLU activation) alternating with max pooling layers (pool size = 2), followed by a flatten layer, dropout layer (rate = 0.2), and two fully connected layers (64 units with ReLU activation and 1 unit with linear activation for pH prediction).

**Figure S2:** Correlation of pHe measurements between Bloch fitting and ML methods using scatter plots. These plots show pHe distributions for training (left column) and testing (right column) datasets using (A, B) RFR using CEST spectra and T_1_, T_2_, B_1_, B_0_ maps; (C, D) RFR using only CEST spectra; (E, F) CNN using only CEST spectra. This graph shows all 4129 datapoints, although the majority of these datapoints are overlapped. For comparison, Figure 3 shows ranges of pHe values from Bloch fitting. The dashed lines represent the line of identity.

**Figure S3:** Feature importance from the RFR models. Feature importance for the RFR model using (A) CEST spectra with T_1_, T_2_, B_1_, and B_0_ maps, and (B) CEST spectra alone. Importance is broadly distributed across the Z‐spectrum in both models rather than concentrated at the amide‐proton CEST peaks conventionally used for pH quantification (4.2, 5.6 ppm; red bars). Auxiliary MRI parameters (T_1_, T_2_, B_1_, B_0_; gray bars) rank among the highest‐importance features in (A) despite providing no measurable improvement in predictive accuracy relative to the CEST‐only model (R^2^ = 0.997 between the two RFR models, Figure 5A), suggesting this information is already encoded in the shape of the raw Z‐spectrum.

## Data Availability

Our analysis methods, MR images, and data are available in GitHub (https://github.com/CAMEL‐MartyPagel/acidoCEST_MachineLearning).
