# Supplementary material for: A Machine Learning Approach That Measures Extracellular pH Within an In Vivo Tumor Model Using acidoCEST MRI
Source: NMR Biomed. 2026 Aug 26;39(10):e70379. doi: 10.1002/nbm.70379 (PMC13508042; doi:10.1002/nbm.70379)
Supplement: Supplementary file 1 [file NBM-39-e70379-s003.pdf]

<u>**Supplementary Material**</u>

A machine learning approach that measures extracellular pH within an in vivo tumor model using acidoCEST MRI

Alia Khaled, Chetan B. Dhakan, Lucas B. McCullum, Jorge de la Cerda,
F. William Schuler, Tianzhe Li, Mark D. Pagel

1. Introduction

The supplementary materials consist of a list of the acquisition parameters for MRI acquisitions, three supplementary figures, and one supplementary table. Citations are numbered according to the reference list provided in the main document.

The supplementary figure shows a graph containing all 4,129 datapoints used in the machine learning methods. The large majority of these 4,129 datapoints are overlapped, which makes the distributions of data difficult to view in each graph. For comparison, Figure 3 shows the same relationships after we binned ranges of the 4,129 datapoints into intervals of 0.17 pHe units. Figure 3 more clearly shows the distributions of data in each graph by using a box and whiskers format for each bin.

2.0 MRI acquisition parameters

Anatomical MRI scans were acquired with a Rapid Acquisition with Relaxation Enhancement (RARE) acquisition method using the following parameters:

- TR: 1000 ms
- TE: 43.36 ms
- RARE factor: 16
- excitation angle: 15°
- matrix: 128 × 128
- field of view: 38.4 × 38.4 mm²
- In-plane resolution: 300 x 300 μm
- slice thickness: 1 mm
- Number of contiguous slices: 40 or 1

T_1 maps were acquired using rapid acquisition with relaxation enhancement and variable time of recovery (RAREVTR) acquisition.[25]

- TR: 150.0, 302.8, 483.3, 703.8, 986.9, 1383.4, 2050.0, and 5000 ms
- TE: 21.68 ms
- RARE factor: 8
- excitation angle: 90°
- matrix: 64x64
- field of view: 3.84 cm × 3.84 cm
- in-plane resolution: 600 μm × 600 μm
- slice thickness: 1 mm
- number of slices: 1

T2 maps were acquired using a multi-slice multi-echo (MSME) acquisition.[26]

- TR: 1006.655 ms
- TE: 6.0 ms

- Number of echos: 150
- excitation angle: 90°
- matrix: 64x64
- field of view: 3.84 cm × 3.84 cm
- in-plane resolution: 600 μm × 600 μm
- slice thickness: 1 mm
- number of slices: 1

B_1 maps were acquired using the double-angle method with a single gradient echo sequence with excitation flip angles of 30° and 60° and complete relaxation between excitations.[27]
- TR: 10000 ms
- TE: 43.36 ms
- excitation angle: 30° or 60°
- matrix: 64x64
- field of view: 3.84 cm × 3.84 cm
- in-plane resolution: 600 μm × 600 μm
- slice thickness: 1 mm
- number of slices: 1

B_0 maps were acquired using the water saturation shift referencing (WASSR) method.[28]
- TR: 2.67 ms
- TE: 1.335 ms
- excitation angle: 15°
- linear encoding order
- unbalanced 'FID' mode
- matrix: 64x64
- field of view: 3.84 cm × 3.84 cm
- in-plane resolution: 600 μm × 600 μm
- slice thickness: 1 mm
- number of slices: 1
- saturation power: 1 μT
- saturation time: 0.5 s
- number of saturation frequences: 45
- Saturation frequency list: -100 ppm followed by -1 to 1 ppm with a 0.05 ppm interval

No additional spoiling or fat saturation pulses were applied between the saturation and the acquisition.

The acidoCEST MRI scans were performed with a Fast Imaging with Steady state Precession (FISP) acquisition method that was prepended with selective saturation.[29] The FISP acquisition parameters were as follows:
- TR: 2.67 ms
- TE: 1.335 ms
- excitation angle: 15°
- linear encoding order
- unbalanced 'FID' mode
- matrix: 64x64
- field of view: 3.84 cm × 3.84 cm
- in-plane resolution: 600 μm × 600 μm
- slice thickness: 1 mm

- number of slices: 1

Prior to FISP acquisition, the CEST saturation was achieved with a single square-shaped RF pulse with the following parameters:

- saturation time: 3 s
- saturation power: 3.5 μT
- number of saturation frequences: 45
- Saturation frequency list: -100, -100, -100, -10, -9, -8, -7, -6, -5, -4, -3, -2, -1.5, -1, -0.5, 0, 0.5, 1, 1.5, 2, 2.5, 3, 3.25, 3.5, 3.75, 4, 4.25, 4.5, 4.75, 5, 5.25, 5.5, 5.75, 6, 6.25, 6.5, 6.75, 7, 7.5, 8, 8.5, 9, 9.5, 10 ppm

No additional spoiling or fat saturation pulses were applied between the saturation and the acquisition.

We fit the data from T_1 and T_2 mapping experiments with a mono-exponential model using MATLAB R2022b (The Mathworks, Inc., Natick, MA, USA) and adopted the T_1 and T_2 values with a smaller coefficient of variation for each sample tube and for each mouse. We fit the data from B_0 mapping experiments with a Lorentzian line shape model to estimate the chemical shift value with the minimum signal. We fit the data from B_1 mapping experiments using Equation S1.

$$\%B_1 = \{(A_{60} / 2{*}A_{30}) * (360/2\pi) * (100/30) \qquad [S1]$$

where
$\%B_1$: the percentage of B1 power relative to the expected B1 power
A_{60}: Signal amplitude with a 60° excitation angle
A_{30}: Signal amplitude with a 30° excitation angle
($360/2\pi$): A conversion from radians to degrees
(100/30): a conversion from an angle in degrees to a % relative to 30°

3.0 Bloch Fitting method

To perform our Bloch fitting method, we first applied Gaussian spatial smoothing to the MR images. Then we obtained the average of the four pre-injection CEST MR image sets. For each pixel, we obtained the MR signal amplitude at each saturation frequency, normalized the amplitudes by setting the average of the three amplitudes at -100 ppm saturation frequency to 100%, and then plotted the normalized signal amplitude vs. saturation frequency to generate a pre-injection CEST spectrum. We repeated this process for the six post-injection CEST MR image sets to generate a post-injection CEST spectrum. We subtracted the pre-injection CEST spectrum from the post-injection CEST spectrum to isolate the signal from iopamidol. We did not apply spectral smoothing.

We fit this difference CEST spectrum for each pixel using our customized software for Bloch fitting (Matlab R2023a, The Mathworks, Natick, MA), which is publicly available at available at https://github.com/CAMEL-MartyPagel/acidoCEST_MRI_Matlab. To develop this software, we modified the standard Bloch-McConnell equations by substituting pHe for the pH-dependent chemical exchange rates of iopamidol. We iteratively fit modified Bloch-McConnell equations to the CEST spectrum of each pixel within the tumor ROI. This fitting process directly results in a pHe value from the Bloch fitting process.

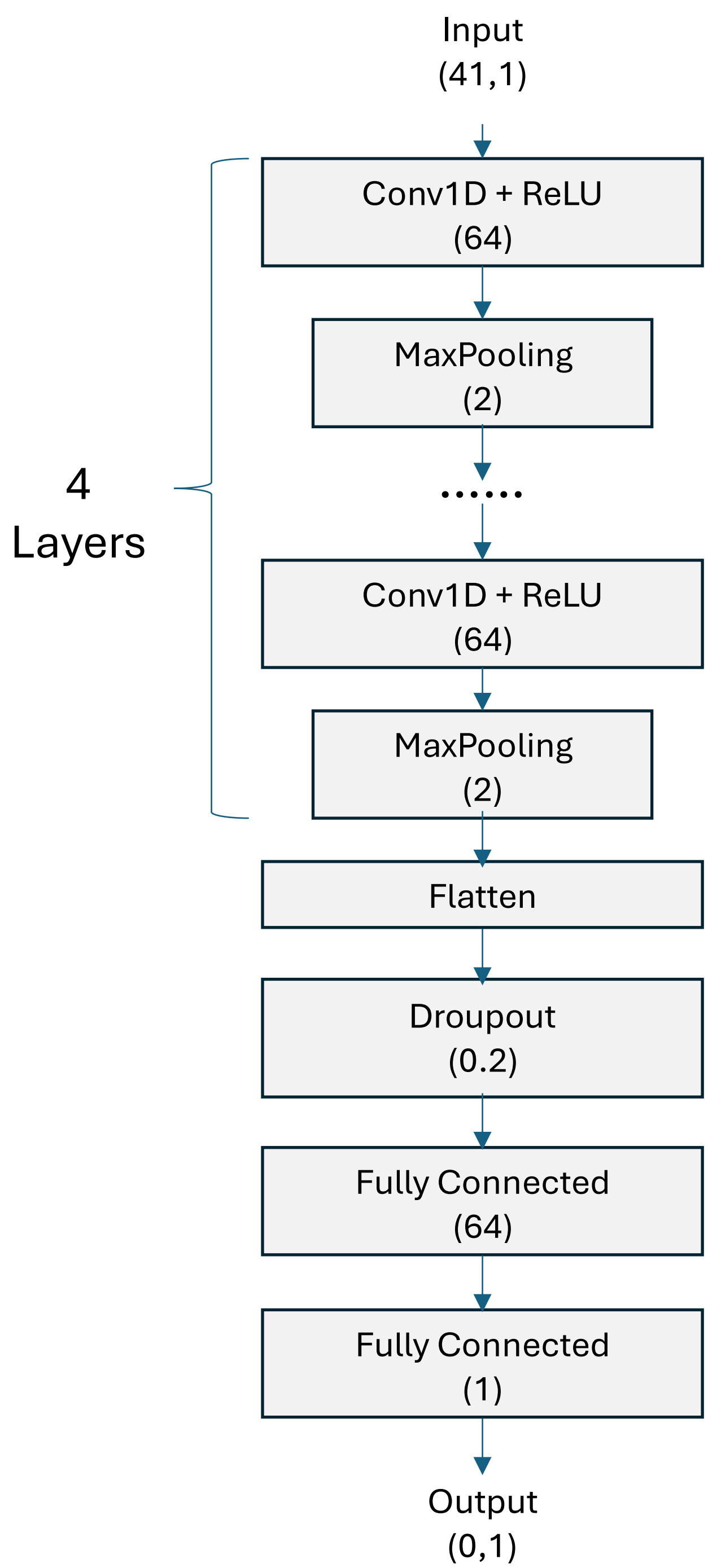

Supplementary Figure S1. CNN architecture for acidoCEST MRI analysis. The 1D convolutional neural network consists of four convolutional layers (each with 64 filters, kernel size = 3, and ReLU activation) alternating with max pooling layers (pool size = 2), followed by a flatten layer, dropout layer (rate = 0.2), and two fully connected layers (64 units with ReLU activation and 1 unit with linear activation for pH prediction).

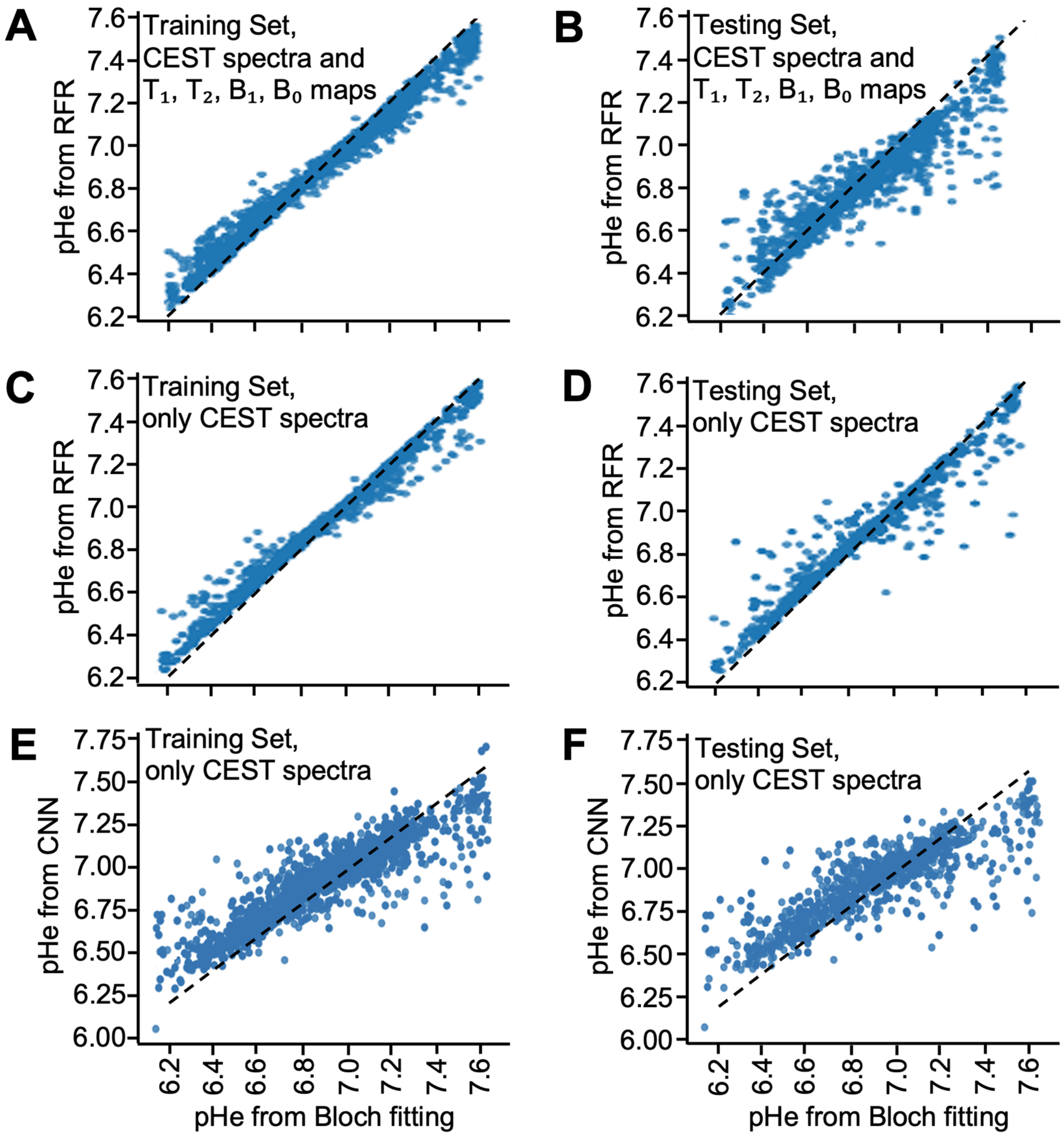

Supplementary Figure S2. Correlation of pHe measurements between Bloch fitting and machine learning methods using scatter plots. These plots show pHe distributions for training (left column) and testing (right column) datasets using A,B) RFR using CEST spectra and T_1, T_2, B_1, B_0 maps; C,D) RFR using only CEST spectra; E,F) CNN using only CEST spectra. This graph shows all 4129 datapoints. The majority of these datapoints are overlapped, making it difficult to appreciate the distribution of data. For comparison, Figure 3 shows ranges of pHe values from Bloch fitting that improves the visualization of the distributions. The dashed lines represent the line of identity.

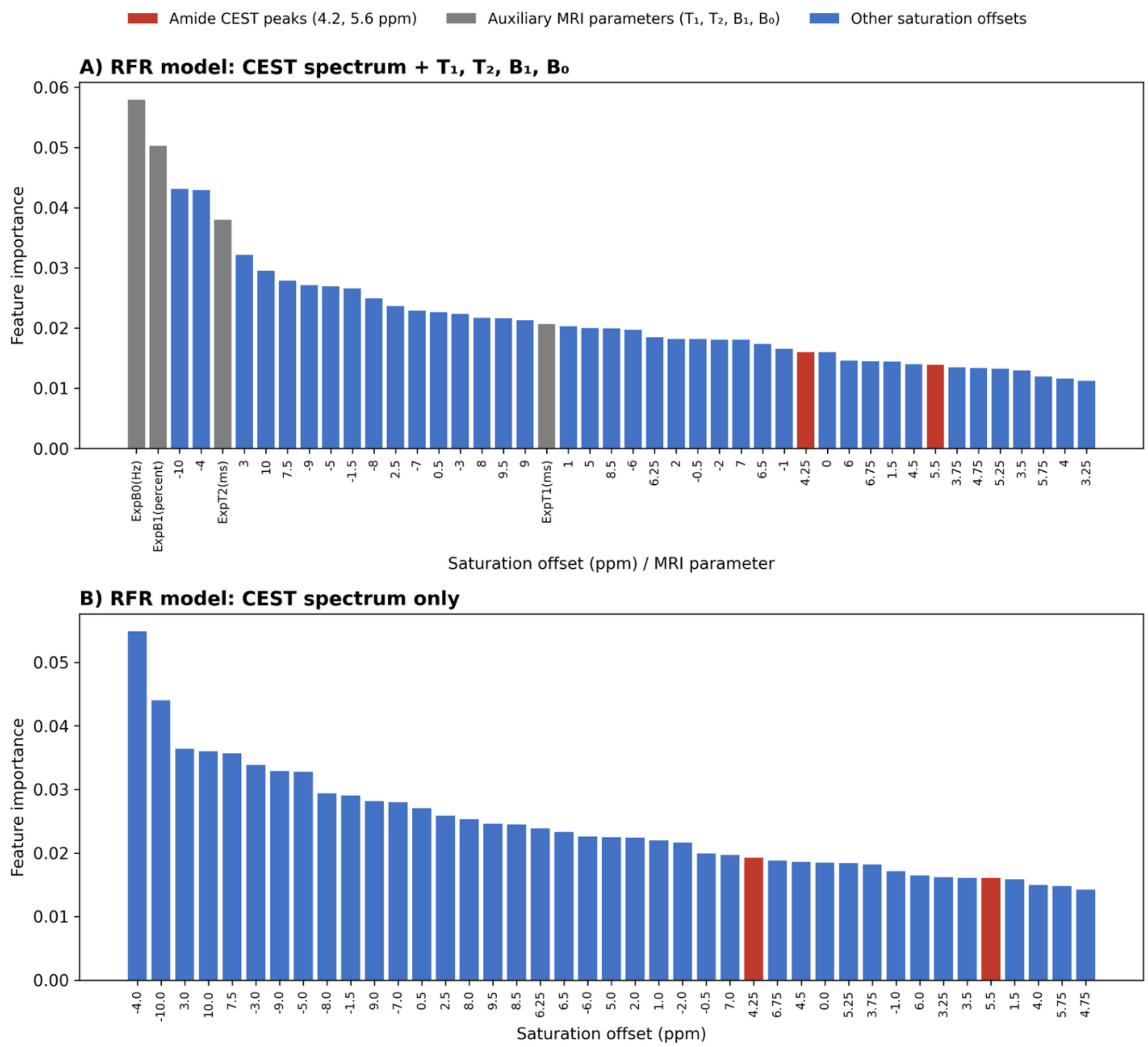

Supplementary Figure S3. Feature importance from the RFR models. Feature importance for the RFR model using (A) CEST spectra with T1, T2, B1, and B0 maps, and (B) CEST spectra alone. Importance is broadly distributed across the Z-spectrum in both models rather than concentrated at the amide-proton CEST peaks conventionally used for pH quantification (4.2, 5.6 ppm; red bars). Auxiliary MRI parameters (T1, T2, B1, B0; gray bars) rank among the highest-importance features in (A) despite providing no measurable improvement in predictive accuracy relative to the CEST-only model (R^2 = 0.997 between the two RFR models, Fig. 5A), suggesting this information is already encoded in the shape of the raw Z-spectrum.

Supplementary Table S1. Permutation (label-shuffling) test for the RFR models. Each RFR model was retrained with identical hyperparameters and features, but with pHe labels randomly permuted across training pixels prior to fitting ("Permuted" rows), and evaluated against the true, unpermuted test-set labels. Both permuted models performed substantially worse than the corresponding trained models ("Real" rows), including negative R^2 values (worse than predicting the mean pHe value), confirming that the trained models' accuracy reflects a genuine learned relationship between CEST spectral features and pHe rather than chance association or overfitting.

Model	Labels	MAPE (test)	R^2 (test)	pH-unit MAE (test)
RFR, CEST only	Real	0.79%	0.897	0.054
RFR, CEST only	Permuted	4.16%	−0.343	0.286
RFR, all features	Real	1.37%	0.800	0.094
RFR, all features	Permuted	3.86%	−0.145	0.266
